# Mechanisms controlling the trafficking, localization, and abundance of presynaptic Ca^2+^ channels

**DOI:** 10.3389/fnmol.2022.1116729

**Published:** 2023-01-13

**Authors:** Karen L. Cunningham, J. Troy Littleton

**Affiliations:** The Picower Institute for Learning and Memory, Department of Biology, Department of Brain and Cognitive Sciences, Massachusetts Institute of Technology, Cambridge, MA, United States

**Keywords:** voltage-gated calcium channel, synapse, active zone, neurotransmitter release, protein trafficking

## Abstract

Voltage-gated Ca^2+^ channels (VGCCs) mediate Ca^2+^ influx to trigger neurotransmitter release at specialized presynaptic sites termed active zones (AZs). The abundance of VGCCs at AZs regulates neurotransmitter release probability (*P_r_*), a key presynaptic determinant of synaptic strength. Given this functional significance, defining the processes that cooperate to establish AZ VGCC abundance is critical for understanding how these mechanisms set synaptic strength and how they might be regulated to control presynaptic plasticity. VGCC abundance at AZs involves multiple steps, including channel biosynthesis (transcription, translation, and trafficking through the endomembrane system), forward axonal trafficking and delivery to synaptic terminals, incorporation and retention at presynaptic sites, and protein recycling. Here we discuss mechanisms that control VGCC abundance at synapses, highlighting findings from invertebrate and vertebrate models.

## Introduction

Electrical signaling within the nervous system provides a fast and robust mechanism for transmitting action potentials to synaptic terminals. Voltage-gated calcium channels (VGCCs) are essential for translating electrical propagation of action potentials into intracellular chemical signals. When the membrane voltage passes a critical threshold, VGCCs open and allow influx of Ca^2+^ ions into the cell. Baseline Ca^2+^ concentrations in the cytosol are kept extremely low through extensive buffering and fast extrusion *via* pumps, allowing Ca^2+^ to act as a potent intracellular signal to regulate a diversity of processes, such as vesicle fusion, phosphorylation, or transcriptional changes (Berridge et al., 2003). At chemical synapses, presynaptic VGCCs trigger neurotransmitter release from synaptic vesicles (SVs) by mediating Ca^2+^ influx, which activates the SV protein Synaptotagmin 1 (Syt1) to drive fusion of the SV and plasma membranes (Littleton et al., 1993, 1994; DiAntonio and Schwarz, 1994; Sauvola and Littleton, 2021).

**Figure 1:**
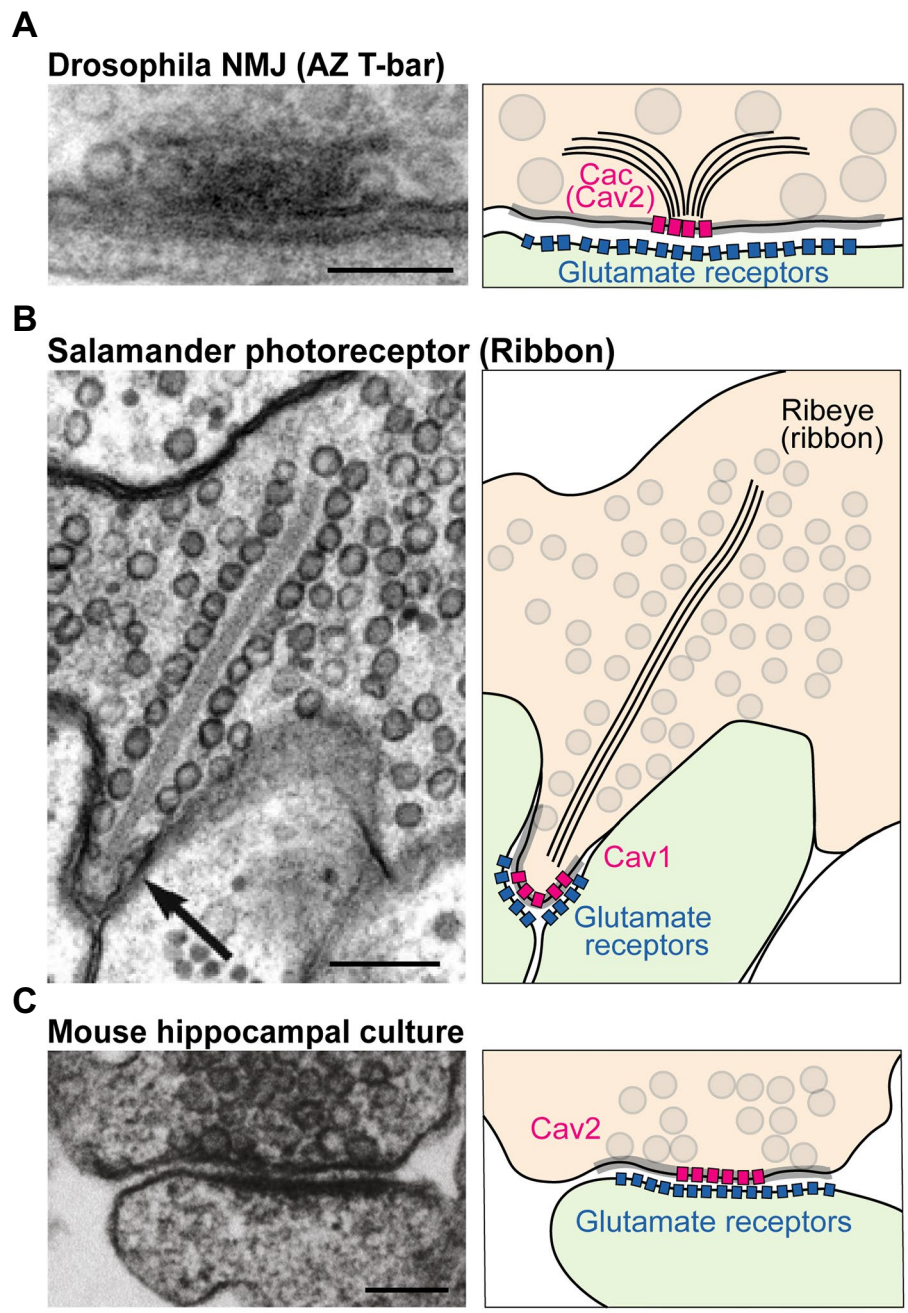
Structure and molecular composition of AZs. **(A)** Left: EM of an AZ at the *Drosophila* NMJ (modified from Fouquet et al., 2009; scale bar: 100 nm). Right: molecular depiction of the AZ. The presynaptic terminal is colored in orange, and the postsynaptic cell green. Gray circles represent SVs. The gray highlighted zone along the presynaptic membrane marks the AZ area. Bruchpilot (BRP; black lines) forms the “T-bar” structure together with other scaffolds (not shown). The presynaptic Ca_v_2 channel Cacophony (Cac) clusters at the base of the T-bar, while Glutamate Receptors cluster postsynaptically. **(B)** EM (modified from Thoreson et al., 2004; scale bar: 200 nm) and depiction of a salamander photoreceptor ribbon synapse. The ribbon is an electron dense projection (formed by the protein Ribeye) and is lined with SVs. Ca_v_1 family channels mediate fusion at this synapse. **(C)** EM (modified from Kaeser et al., 2011; scale bar: 100 nm) and model of a mouse hippocampal cultured synapse. SV fusion at mammalian CNS neurons is primarily supported by Ca_v_2.1 and Ca_v_2.2 channels (pink).

Presynaptic neurotransmitter release lags behind intracellular Ca^2+^ influx by less than a millisecond (Katz and Miledi, 1965; Borst and Sakmann, 1996; Sabatini and Regehr, 1996; Neher, 1998). This incredible speed reflects the tight spatial organization of fusion-primed SVs near VGCCs. This spatial coordination occurs at active zones (AZs), specialized domains within the presynaptic membrane where a macromolecular complex of conserved scaffold proteins recruits SVs to clusters of VGCCs for efficient Ca^2+^ use (Kawasaki et al., 2004; Bucurenciu et al., 2008; Catterall and Few, 2008; Wang et al., 2008; Fouquet et al., 2009; Eggermann et al., 2011; Chen et al., 2015; Nakamura et al., 2015). Although the opening of a single VGCC can trigger SV release at some synapses, VGCCs are typically clustered at AZs to produce a larger transient domain of intracellular Ca^2+^ (Jarsky et al., 2010; Bartoletti et al., 2011; Nakamura et al., 2015). AZs in different neurons and species differ in their ultrastructure. For example, AZs at the *Drosophila* neuromuscular junction (NMJ) show an electron dense “T-bar” structure composed primarily of the scaffold protein Bruchpilot (BRP) when viewed by EM (Figure 1A; Fouquet et al., 2009). In contrast, sensory synapses within photoreceptors and hair cells contain a long synaptic ribbon that is predicted to facilitate robust SV release at these terminals (Figure 1B). Mammalian CNS synapses lack such a striking dense projection but still have an increased electron density that corresponds to the dense network of AZ scaffold proteins (Figure 1C). Despite these ultrastructural differences, scaffolding proteins present at synapses are generally conserved. The major scaffolds that cluster VGCCs at AZs are Rab3-interacting molecules (RIMs), RIM-binding proteins (RBPs), ELKS/CAST, and Bassoon/Piccolo/Fife (Kittel et al., 2006; Müller et al., 2010; Han et al., 2011; Kaeser et al., 2011; Liu et al., 2011; Graf et al., 2012; Kiyonaka et al., 2012; Davydova et al., 2014; Bruckner et al., 2017; Xuan et al., 2017). These proteins form multiple binding interactions with VGCCs to provide functional redundancy in VGCC clustering at AZs.

**Figure 2:**
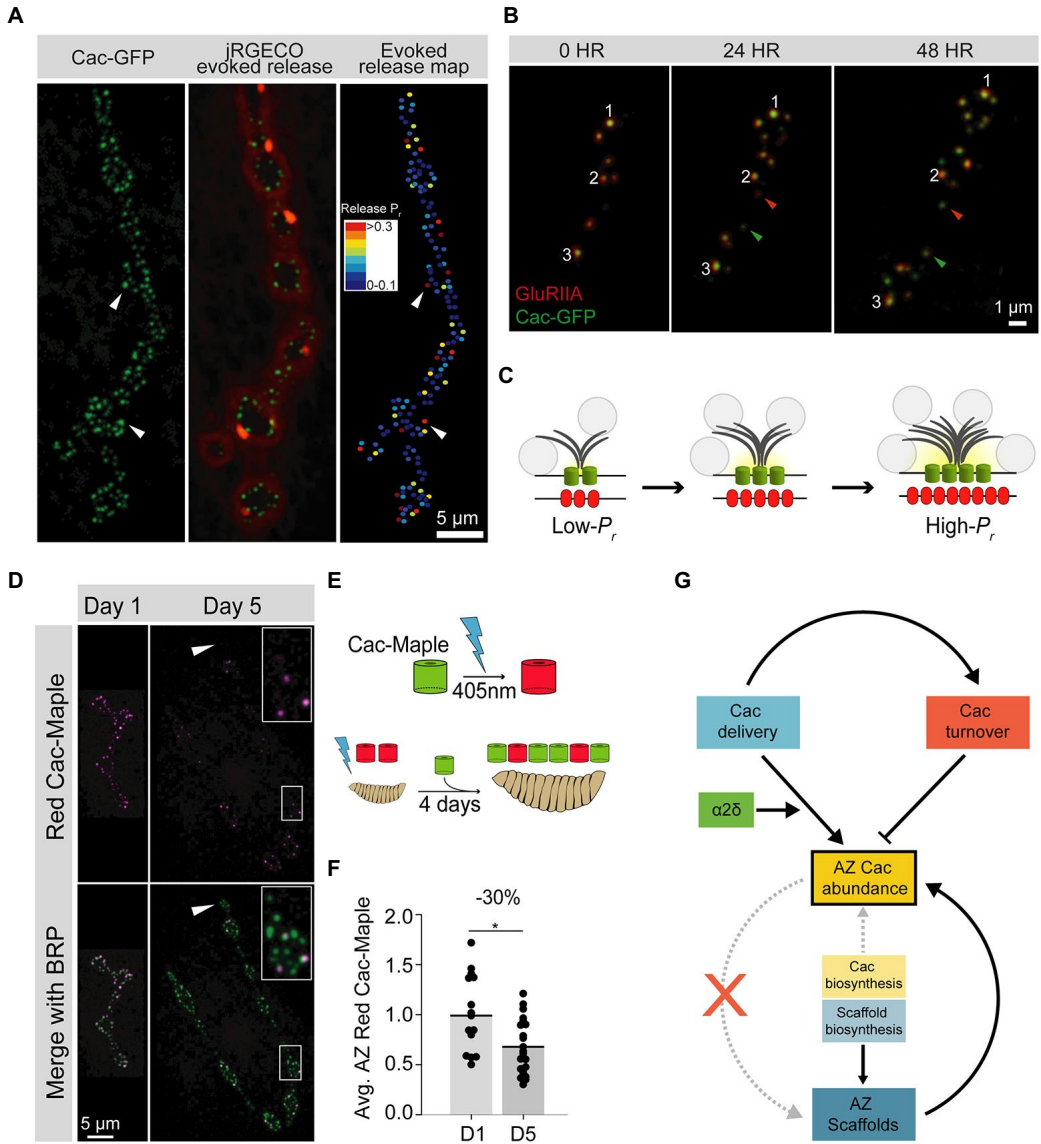
Cac regulation at the Drosophila third instar larval NMJ. **(A)** Representative image of a *Drosophila* larval NMJ. AZs are marked by clusters of Cac-GFP (green), and the jRGECO calcium sensor (red) is tethered to the postsynaptic membrane and indicates evoked release events at individual AZs. An evoked release heat map (right); generated from videos of jRGECO responses following a series of individual APs indicates *P_r_* for each AZ, with high-*P_r_* AZs in red and low-*P_r_* sites in blue. **(B)** Sequential intravital imaging of a growing NMJ in an intact, anesthetized animal. Glutamate Receptor subunit GluRIIA-RFP marks postsynaptic densities and Cac-GFP marks presynaptic AZs. Arrows mark several new AZs formed after the 0-h timepoint, and white numbers track individual AZs through the image series. **(C)** Schematic of an AZ as it structurally and functionally matures from a young (low-*P_r_*) site to an old (high-*P_r_*) site through the multi-day acquisition of key components including BRP (gray), and Cac (green). Postsynaptic Glutamate Receptor abundance (red) also increases throughout maturation. SVs are marked as gray circles. **(D)** Representative images of Cac-Maple (magenta) and the AZ scaffold BRP (green) at AZs of the *Drosophila* NMJ, 1 day or 5 days after complete photoconversion. White arrows mark a bouton that formed after photoconversion, and is completely devoid of red Cac-Maple channels. The bouton outlined in white is enlarged in the upper right corner of each Day 5 image. **(E)** Schematic of Cac-Maple photoconversion (top panel) and experimental approach to measuring Cac turnover rate at AZs (bottom panel). Cac-Maple is green, and photoconverts permanently to red upon illumination with a 405 nm light (blue lightning bolt). Photoconversion of an entire first instar larva, followed by 4 days of growth (during which time new green Cac-Maple channels are added to growing NMJs) results in a mixed pool of AZs with some AZs having only green channels (those that were added to the NMJ after the photoconversion event) and some AZs having red signal as well (representing channels present at the initial photoconversion timepoint). **(F)** Quantification of average red Cac-Maple abundance per AZ 1 day and 5 days post-photoconversion. A 30% reduction in red Cac-Maple levels occurs over this 4-day window. **(G)** Model of VGCC (Cac) regulation at the *Drosophila* NMJ. Cac delivery and turnover rates can be measured *in vivo* at this synapse. Both delivery (blue) and turnover (orange) cooperate to establish AZ Cac abundance. The α2δ subunit (green) positively regulates Cac delivery. AZ Cac abundance is only weakly regulated by Cac biosynthesis levels, as AZ Cac levels are highly buffered against changes in biosynthesis (yellow). In contrast, AZ scaffold biosynthesis plays a larger role in regulating AZ levels of the scaffold protein BRP (blue). While the presence of the AZ scaffold BRP is required for proper AZ Cac abundance (upward curved solid arrow), AZ Cac is not required for scaffold formation (red X). Figure panels **(A,B)** were adapted from Akbergenova et al. (2018). Figure panels **(D,G)** were adapted from Cunningham et al. (2022).

Although AZs are specialized for SV fusion, not every AZ releases a SV following an action potential. Instead, neurons display a wide range of synaptic efficacies. Synaptic strength is a composite of both pre- and post-synaptic factors, and its regulation increases diversity for supporting circuit function and plasticity (Atwood and Karunanithi, 2002). A key presynaptic determinant of synaptic strength is neurotransmitter release probability (*P_r_*), the likelihood of SV fusion after an action potential. Evoked *P_r_* is partially regulated at the AZ-level and can vary dramatically across AZs formed by a single neuron (Figure 2A; Peled and Isacoff, 2011; Melom et al., 2013; Akbergenova et al., 2018; Sauvola et al., 2021; Newman et al., 2022). The amount of presynaptic Ca^2+^ influx at AZs, regulated in large part by the number of VGCCs clustered at the AZ, is a major determinant of *P_r_* (Augustine et al., 1985; Borst and Sakmann, 1996; Wang et al., 2008; Bartoletti et al., 2011; Ariel et al., 2012; Sheng et al., 2012; Akbergenova et al., 2018; Newman et al., 2022). As such, VGCC function, regulation, and localization is central to how neurons control presynaptic output.

This review explores current models for how VGCC abundance is regulated at presynaptic AZs, with an emphasis on Ca_v_2 family channels, which are the primary mediators of neurotransmission at most synapses (Dolphin and Lee, 2020). We focus exclusively on processes that mediate channel localization and abundance, as the structure and function of the channel has been extensively reviewed elsewhere (Catterall et al., 2020). We examine the regulation of VGCC localization at all stages of the channel’s life, beginning with biosynthesis and progression through the ER and Golgi that requires auxiliary subunits. After axonal trafficking to synaptic terminals, channels are stabilized at AZs through multiple binding interactions with conserved AZ scaffold proteins including ELKS/CAST, RIM and RBP. We review evidence for the “slot” model of VGCC AZ abundance that suggests excess VGCCs compete for limited AZ localization through rate-limiting binding interactions downstream of channel biosynthesis (Cao et al., 2004; Cao and Tsien, 2010). Next, we focus on the channel’s mobility once incorporated into AZs. Despite binding to multiple scaffolds, single-molecule tracking studies indicate VGCCs are highly mobile within the AZ (Mercer et al., 2011, 2012; Thoreson et al., 2013; Schneider et al., 2015; Voigt et al., 2016; Ghelani et al., 2022). In addition to VGCC mobility, we review molecular pathways facilitating endocytosis, although limited *in situ* information is available to contextualize these molecular pathways in intact circuits. Finally, we discuss how these processes are regulated during synaptic plasticity. Each of these steps—biosynthesis, trafficking, AZ scaffold-binding, mobility, and turnover, provide points of VGCC regulation that can be modulated to control presynaptic *P_r_*.

## The VGCC is a multisubunit complex

**Figure 3:**
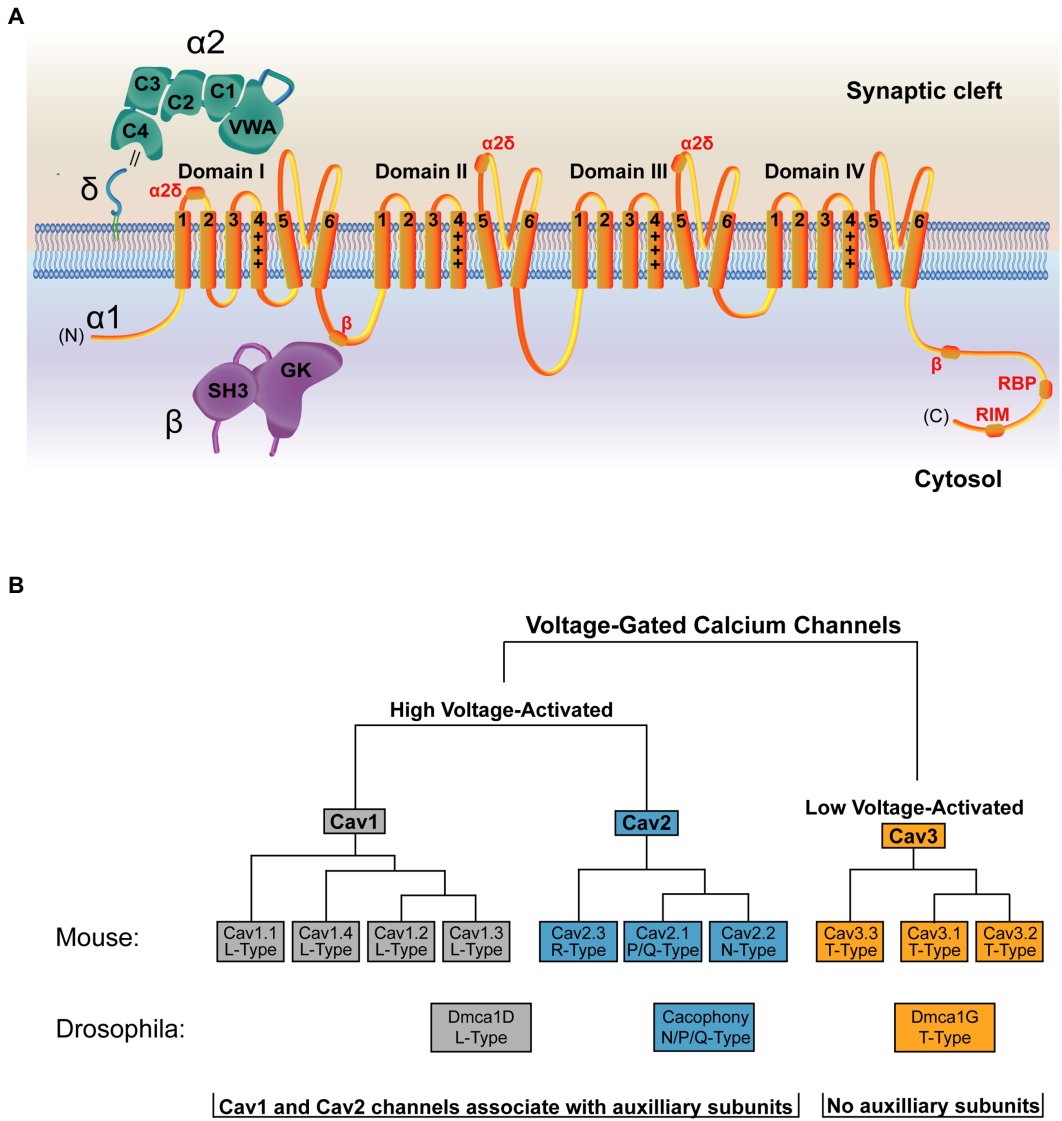
Structure and organization of the VGCC family. **(A)** Structure of a VGCC complex with the α1 pore-forming subunit in orange. VGCCs have four domains with six transmembrane segments each. Transmembrane segments I-IV comprise the voltage-sensing module, with transmembrane segment IV (marked +++) as the voltage sensing segment. Transmembrane segments V and VI form the ion-conducting pore. The cytoplasmic C-terminal tail interacts with multiple binding partners, including a secondary Cavß interaction site (Walker et al., 1998) and binding regions for RBP and RIM (Lübbert et al., 2017). The α2δ subunit (green) is extracellular and comprised of α2 and δ peptides linked *via* a disulfide bond (double black line) and anchored to the outer membrane leaflet *via* a lipid anchor (Davies et al., 2010; Wu et al., 2015). α2δ contains five domains, with the Von Willebrand Factor-A (VWA) and the first two Cache domains (C1 and C2) interacting with α1 (Wu et al., 2016). Sites of α2δ-interaction on the α1 subunit are marked in red. The Cavß subunit (purple) is cytosolic and comprised of an SH3 domain and a Guanylate Kinase (GK) domain. The primary α1 interaction site is mediated through an intracellular loop in domain I of the channel and the Cavß GK domain (Pragnell et al., 1994; Cantí et al., 2001; Chen et al., 2004; Opatowsky et al., 2004; Van Petegem et al., 2004; Wu et al., 2016). **(B)** The mammalian VGCC family is comprised of 7 high voltage-activated VGCCs (Ca_v_1 and Ca_v_2 family channels) and 3 low voltage-activated VGCCs (Ca_v_3 family channels). *Drosophila* (shown below the mammalian tree) encodes one VGCC per class, with Dmca1D encoding the sole L-Type Ca_v_1 channel (Eberl et al., 1998; Hara et al., 2015), Cacophony (Cac) encoding the sole N/Q/P type Ca_v_2 channel that mediates synaptic transmission (Smith et al., 1996; Kawasaki et al., 2000, 2004), and Dmca1G encoding the sole T-Type Ca_v_3 channel (Jeong et al., 2015). Ca_v_1 and Ca_v_2 channels associate with auxiliary subunits, while Ca_v_3 channels do not.

High resolution structures are available of the VGCC and its auxiliary subunits (Wu et al., 2015, 2016). Like other ion channels, VGCCs have a pore-forming α1 subunit that selectively fluxes Ca^2+^, as well as several auxiliary subunits that regulate channel trafficking, stabilization, and function. The α1 subunit of the VGCC is closely related to voltage-gated sodium (Na_v_) channels, and only several amino acid changes in the pore region are required to convert a Na_v_ channel into one capable of fluxing Ca^2+^ (Tang et al., 2014). The VGCC α1 subunit contains four domains, each with six transmembrane spanning segments. Transmembrane segments I–IV comprise the voltage-sensing module of the channel, while segments V and VI form the Ca^2+^ selective pore (Figure 3A; Catterall et al., 2020). Despite conservation of α1 structure with other voltage-gated ion channels, the set of auxiliary subunits that regulates VGCCs are unique in the voltage-gated ion channel superfamily (Witcher et al., 1993; Yu et al., 2005). An extracellular α2δ subunit facilitates forward trafficking of the VGCC and modulates its gating and conductance properties (Dolphin, 2016). In addition, a cytosolic ß subunit acts as a chaperone during biosynthesis and is required for VGCC membrane expression (Figure 3A, Pragnell et al., 1994; Gregg et al., 1996; Cantí et al., 2001; Van Petegem et al., 2004; Weissgerber et al., 2006; Altier et al., 2011; Waithe et al., 2011; Dolphin, 2016). These subunits also support interactions between the channel and its signaling or scaffolding partners (Müller et al., 2010).

Mammals encode three VGCC families (Ca_v_1–Ca_v_3) defined by their pore-forming α1 subunit (Figure 3B; Dolphin, 2016). Of these, the four Ca_v_1 channels (also called L-type based on initial current characterization) and the three Ca_v_2 channels (P/Q-, N-, and R-type) are high-voltage activated and are the dominant contributors to synaptic transmission at presynaptic AZs (Luebke et al., 1993; Takahashi and Momiyama, 1993; Wheeler et al., 1994; Reuter, 1995; Reid et al., 1997; Wu et al., 1999; Dolphin and Lee, 2020). Ca_v_2 channels mediate the majority of neurotransmission in the CNS, while Ca_v_1 channels are important in sensory neurons like inner hair cells and photoreceptors. The three Ca_v_3 channels (T-type) are low-voltage activated, do not play a central role in mediating evoked synaptic transmission, and do not require the canonical auxiliary subunits (Dolphin and Lee, 2020). Invertebrate VGCCs also mediate synaptic transmission but have less redundancy. *Drosophila* encodes one family member from each of the three VGCC families, and the single Ca_v_2 family VGCC (Cacophony; Cac) complexes with a single α2δ family member (Straightjacket) to mediate neurotransmission at synapses (Kawasaki et al., 2004; Ly et al., 2008; Ryglewski et al., 2012; Heinrich and Ryglewski, 2020; Figure 3B; Table 1).

**Table 1:** Summary of AZ structure and VGCC localization/abundance phenotypes in *Mus musculus* (*M. mus*), *Caenorhabditis elegans* (*C. ele*), and *Drosophila melanogaster* (*D. mel*) AZ and VGCC mutants.

Function	Protein Family	Species	Gene name	Phenotype (VGCC)	References
AZ Scaffold	ELKS/CAST	*M. mus*	ELKS, CAST	Variable, from no effect to mild effect on VGCC abundance	Liu et al. (2014); Dong et al. (2018); Radulovic et al. (2020)
		*C. ele*	ELKS	Not required for VGCC clustering or synaptic transmission	Oh et al. (2021); Deken et al. (2005)
		*D. mel*	Bruchpilot (BRP)	*brp* nulls show a major loss of VGCCs, loss of T-bars; loss of channel stabilization, lower channel confinement, and failure to potentiate VGCC abundance during homeostatic plasticity	Kittel et al. (2006); Fouquet et al. (2009); Wagh et al. (2006); Ghelani et al. (2022); Matkovic et al. (2013)
	RIM	*M. mus*	RIM	*rim* nulls have ultrastructurally normal AZs with ~40% reduced Ca_v_2.1 abundance, reduced release, and fewer docked SVs	Han et al. (2011); Kaeser et al. (2011)
		*C. ele*	RIM/Unc-10	Loss of Ca_v_2 channels without ultrastructural changes	Oh et al. (2021); Koushika et al. (2001)
		*D. mel*	RIM	Loss of Ca_v_2 channels without ultrastructural changes, decreased mobility of Ca_v_2	Graf et al. (2012); Ghelani et al. (2022)
	RBP	*M. mus*	RBP	Reduced coupling of VGCCs to SVs, unaltered VGCC properties or abundance at calyx of Held but enhanced VGCC loss in *rim* mutants; 40% reduced Ca_v_1 abundance in hair cell AZs	Acuna et al. (2015, 2016); Krinner et al. (2017)
		*C. ele*	RIMB-1	*rimb-1/rbp* mutants have normal VGCC localization and abundance but *rbp* enhances the loss of VGCCs in *rim* mutants	Kushibiki et al. (2019)
		*D. mel*	RBP	*rbp* mutants show depletion of VGCCs, disorganization of the BRP scaffold, and decreased mobility of Ca_v_2 channels	Liu et al. (2011); Ghelani et al. (2022)
	Bassoon	*M. mus*	Bassoon	Loss of Ca_v_2.1 (but not Ca_v_2.2) at hippocampal synapses, loss of synaptic ribbons and reduction in VGCCs at sensory synapses	Altrock et al. (2003); Dick et al. (2003); Khimich et al. (2005); Frank et al. (2010); Jing et al. (2013); Davydova et al. (2014)
		*C. ele*	n/a		
		*D. mel*	n/a		
	Piccolo	*M. mus*	Piccolo	No reported involvement in VGCC abundance	
		*C. ele*	Clarinet	Piccolo-RIM homolog	Xuan et al. (2017)
		*D. mel*	Fife	Piccolo-RIM homolog, mutants have a modest reduction in VGCC abundance and reduced VGCC/SV coupling	Bruckner et al. (2017)
VGCC α1 subunits	Ca_v_1 channels (high voltage activated)	*M. mus*	Ca_v_1.1-Ca_v_1.4 (L-type)	Required for synaptic transmission and ribbon stabilization in mammalian sensory synapses	Liu et al. (2013); Zabouri and Haverkamp (2013); Maddox et al. (2020)
		*C. ele*	egl-19 (L-type)	Muscle excitation and contraction, mechanosensation	Frøkjær-Jensen et al. (2006), Lainé et al. (2011), Lee et al. (1997)
		*D. mel*	Dmca1D (L-type)	Essential for viability, muscle calcium currents	Eberl et al. (1998); Hara et al. (2015)
	Ca_v_2 channels (high voltage activated)	*M. mus*	Ca_v_2.1 (P/Q-type) Ca_v_2.2 (N-type) Ca_v_2.3 (R-type)	Supports most neurotransmission in mammalian CNS. Triple conditional knockout of all Ca_v_2s nearly abolishes evoked transmission without impacting AZ number or structure in CNS	Held et al. (2020)
		*C. ele*	Unc-2 (N/P/Q)	N/P/Q related channel required for evoked synaptic transmission	Schafer and Kenyon (1995); Mathews et al. (2003)
		*D. mel*	Cacophony (N/P/Q)	Sole VGCC responsible for evoked synaptic transmission	Smith et al. (1996); Kawasaki et al. (2000, 2004)
	Ca_v_3 channels (low voltage activated)	*M. mus*	Ca_v_3.1-Ca_v_3.3. (T-type)	Mediates low threshold calcium currents in many excitable cell types	Lambert et al. (2014)
		*C. ele*	CCA-1 (T-type)	Muscle contraction	Shtonda and Avery (2005); Steger et al. (2005)
		*D. mel*	Dmca1G (T-type)	Expressed in brain, low voltage activated calcium currents, has a role in regulating sleep	Jeong et al. (2015)
VGCC auxilliary subunits	α2δ	*M. mus*	α2δ_1_- α2δ_4_	α2δ subunits interact with the VGCC and are required for VGCC surface expression in a semi-redundant manner	Kerov et al. (2018); Wang et al. (2017); Schöpf et al. (2021)
		*C. ele*	unc-36	Surface expression of VGCCs	Saheki and Bargmann (2009)
		*D. mel*	Straightjacket (stj), stol	Stj is required for surface expression of presynaptic VGCCs	Dickman et al. (2008); Ly et al. (2008)
	Ca_v_ß	*M. mus*	ß_1_-ß_4_	Required for channel surface expression by preventing proteasomal degradation in the biosynthetic pathway	Katiyar et al. (2015); Ball et al. (2002); Gregg et al. (1996); Weissgerber et al. (2006); Burgess et al. (1997); Obermair et al. (2010)
		*C. ele*	cbb-1	Cbb-1 is essential for viability and voltage dependent calcium currents in muscle	Lainé et al. (2011)
		*D. mel*	Ca-β		Kanamori et al. (2013)

## VGCC auxiliary subunits promote cell surface expression

**Figure 4:**
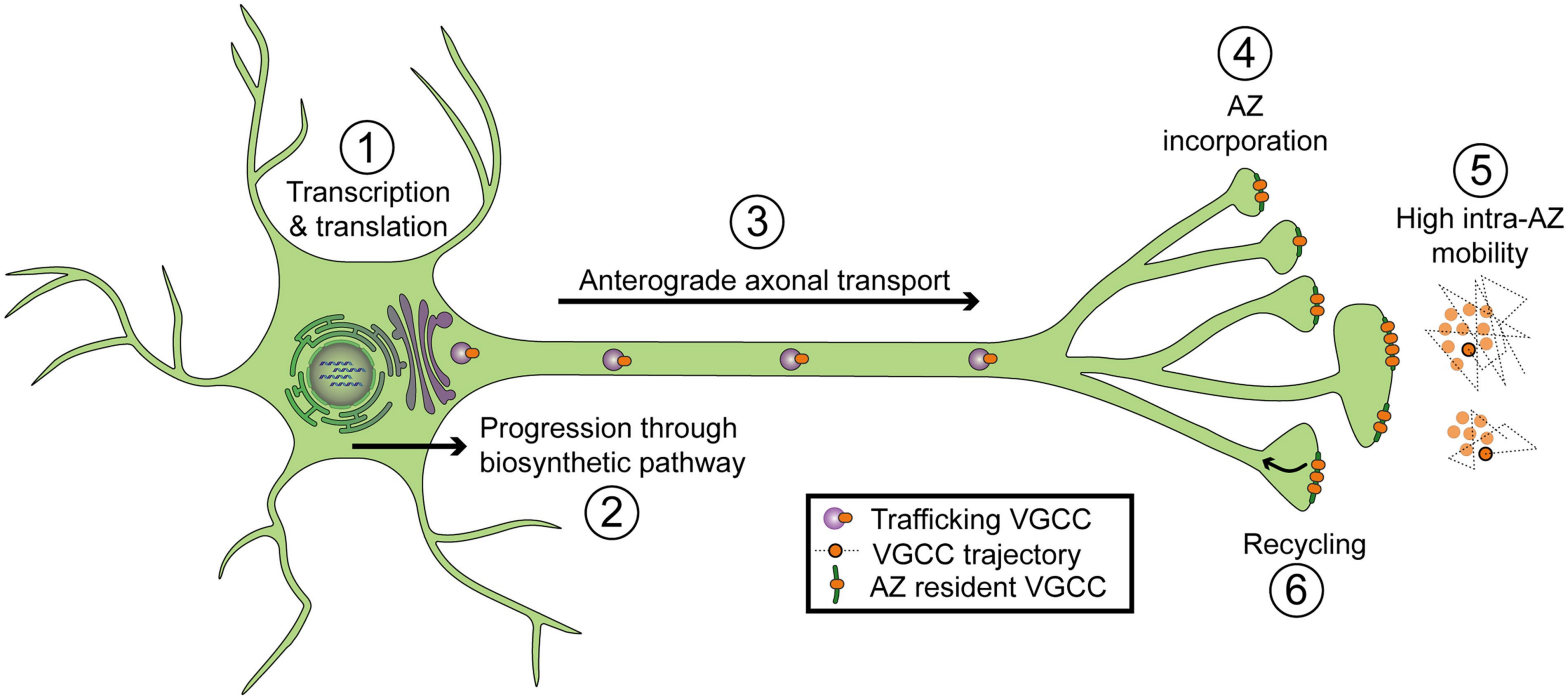
Diagram of regulatory steps involved in setting VGCC abundance at synaptic AZs. (1) Transcription and translation. (2) Progression through the biosynthetic pathway, including the endoplasmic reticulum and Golgi. (3) Forward axonal trafficking. (4) Incorporation into AZs through local interactions with scaffolding proteins. (5) High mobility within individual AZs (intra-AZ mobility) and low inter-AZ mobility. Cartoon on the right depicts a top-down view of VGCCs residing in two nearby AZs. Dotted lines represent short-term trajectories of VGCCs outlined in black. (6) Recycling of VGCCs.

During biosynthesis, VGCCs are translated into the ER and move to the Golgi where they are extensively modified before delivery to the cell surface (Figure 4; step 2). The pore-forming α1 subunit requires co-expression of its auxiliary α2δ and Ca_v_ß subunits to reach the plasma membrane (Brice et al., 1997; Ball et al., 2002; Cassidy et al., 2014). Mammals encode 4 Ca_v_ß genes (ß_1_-ß_4_) that are essential for channel function and result in lethality or severe phenotypes when disrupted (Gregg et al., 1996; Burgess et al., 1997; Weissgerber et al., 2006). Four α2δ subunits (α2δ_1_–α2δ_4_) that are important for survival and display some functional redundancy are also expressed in mammals (Schöpf et al., 2021). While Ca_v_ß is essential for surface expression, α2δ plays a secondary trafficking role that rate-limits presynaptic expression of functional channels. In rodent cultured neurons, overexpression of either α2δ or Ca_v_ß alone dramatically increases presynaptic VGCC abundance, but only α2δ overexpression increases SV fusion (Hoppa et al., 2012). In addition to their requirements in promoting surface expression, these auxiliary subunits play extensive roles in modulating channel properties, including activation, inactivation, and gating, as well as mediating modulation by other regulatory pathways. In addition, α2δ subunits control synapse morphology independent of their role as channel subunits, and can localize to synapses without VGCCs (Kurshan et al., 2009; Dolphin, 2018; Held et al., 2020; Schöpf et al., 2021). These non-localizing or VGCC-independent roles of α2δ and Ca_v_ß subunits have been reviewed elsewhere (Buraei and Yang, 2010; Dolphin, 2018).

Ca_v_ß is a conserved intracellular subunit that controls channel progression through the biosynthetic pathway, determining whether nascent channels are destined for degradation or surface expression. Ca_v_ß contains SH3 and guanylate kinase (GK) domains through which it associates with an intracellular loop between domains I and II of the α1 subunit of Ca_v_1 and Ca_v_2 family VGCCs (Figure 3A; Pragnell et al., 1994; Cantí et al., 2001; Chen et al., 2004; Opatowsky et al., 2004; Van Petegem et al., 2004). Ca_v_ß binding at this site is predicted to promote proper folding of the channel (Opatowsky et al., 2004). In heterologous expression systems, α1 expression alone is insufficient for channel surface expression, but co-expression with Ca_v_ß promotes high levels of surface-expressed α1 (Brice et al., 1997; He et al., 2007; Cassidy et al., 2014). Likewise, *in vivo* knockdown of Ca_v_ß or disruption of the Ca_v_ß binding site on α1 inhibits VGCC surface expression (Berrow et al., 1995; Obermair et al., 2010). Ca_v_1.2 α1 subunits are ubiquitinated and subsequently degraded without Ca_v_ß, and preventing this degradation by pharmacologically blocking the proteasome restores channel surface expression. This data suggests Ca_v_ß is not required for surface expression beyond its role in promoting protein stability in the ER (Altier et al., 2011; Waithe et al., 2011). A secondary Ca_v_ß-binding site is present in the VGCC C-terminal tail (Figure 3A). However structure–function studies at the calyx of Held demonstrated effective rescue of Ca_v_2.1 function by Ca_v_2.1 C-terminal truncation constructs lacking this Ca_v_ß-interaction domain (Walker et al., 1998; Lübbert et al., 2017). Overall these studies indicate Ca_v_ß functions as a chaperone by promoting α1 folding in the ER to prevent degradation.

The conserved VGCC subunit α2δ is also required for VGCC surface expression, though its mechanism of action is less clear. It is entirely extracellular, heavily glycosylated, and anchored to the external leaflet of the presynaptic membrane *via* a glycosyl-phosphatidyl inositol (GPI) anchor (Figure 3A; Davies et al., 2010; Wu et al., 2015). During processing, the α2δ precursor polypeptide is cleaved into α2 and δ subunits that are then linked together *via* disulfide bonds (De Jongh et al., 1990; Jay et al., 1991). Five domains have been identified in α2δ: a Von Willebrand Factor-A (VWA) domain and four Cache domains, with high-resolution structures suggesting multiple interactions between α2δ and the external face of the α1 channel (Figure 3A; Whittaker and Hynes, 2002; Wu et al., 2015, 2016; Cantí et al., 2001). The α2δ subunit can remain associated with the α1 subunit at synapses and modulate channel function, though it is unclear if continued α2δ- α1 interactions are absolutely essential for VGCC activity. Indeed, unlike Ca_v_ß that associates tightly to the α1 subunit with 1:1 stoichiometry, studies have reported a range of interaction strengths between α2δ and α1 ranging from stable to transient association modes (Pragnell et al., 1994; Müller et al., 2010; Cassidy et al., 2014; Voigt et al., 2016). Like Ca_v_ß, α2δ is required for proper surface expression of the channel. While the exact mechanism is unknown, this role of α2δ is likely performed by promoting forward trafficking rather than preventing channel endocytosis (Cantí et al., 2001; Dickman et al., 2008; Ly et al., 2008; Saheki and Bargmann, 2009; Hoppa et al., 2012; Cassidy et al., 2014; Cunningham et al., 2022). Understanding the structure and function of α2δ is of special importance due to its pharmacological targeting by the widely prescribed drugs gabapentin and pregabalin (Gee et al., 1996; Taylor et al., 2007; Eroglu et al., 2009; Dolphin, 2016).

## Axonal trafficking and the “prefabricated synapse” hypothesis

After progression through the biosynthetic pathway, presynaptic VGCCs are trafficked down the axon to synaptic terminals (Figure 4; step 3). VGCC axonal trafficking is largely mysterious, but the trafficking of other presynaptic components such as AZ scaffolds and SV proteins has been extensively studied. Plus-end directed microtubule-based transport mediated by motor proteins of the Kinesin family is the dominant mode of trafficking to terminals (Vale et al., 1996; Goldstein et al., 2008; Hirokawa et al., 2010). Specifically, the anterograde Kinesin-3 family motor Unc-104/KIF1A plays a conserved role in the transport of many synaptic cargoes (Hall and Hedgecock, 1991; Okada et al., 1995; Yonekawa et al., 1998; Pack-Chung et al., 2007; Rivière et al., 2011; Maeder et al., 2014; Zhang et al., 2016, 2017). Kinesin-1 family members have also been implicated in synaptic transport (Gho et al., 1992; Hurd and Saxton, 1996; Gindhart et al., 1998). Since Kinesin diversity alone is insufficient to support the wide need of unique cellular trafficking processes, adaptor proteins that associate with cargo and Kinesins provide additional levels of regulation (Goldstein et al., 2008; Tempes et al., 2020). A conserved adaptor role for the GTPase Arl-8 in supporting Unc-104 mediated synaptic transport was first described in *C. elegans* where SV and AZ proteins are co-transported in an Unc-104/KIF1A dependent pathway (Hall and Hedgecock, 1991; Wu et al., 2013). In a forward genetic screen for genes involved in synapse formation, Arl-8 disruptions were found to deplete SV components from synapses and promote the ectopic accumulation of AZ and SV proteins along axons (Klassen et al., 2010). Likewise, Arl-8 co-traffics with several synaptic proteins, and its loss severely disrupts their synaptic localization as well as synapse growth in *Drosophila* (Vukoja et al., 2018). These studies demonstrate an important and conserved role for Unc-104 and its Arl-8 adaptor in axonal trafficking of synaptic proteins.

Do presynaptic proteins co-transport or arrive independently at developing synapses? Through studies of transport packets containing AZ and SV proteins, several synapse-specific transport organelles have been identified (Goldstein et al., 2008; Vukoja et al., 2018). In mammals, Golgi-derived ~80 nm dense core vesicles called Piccolo-Bassoon transport vesicles (PTVs) traffic many AZ proteins including Piccolo, Bassoon, RIM, CAST, N-Cadherin, Rab3, and Munc18, but lack the SV proteins Syt1 and VAMP (Zhai et al., 2001; Ohtsuka et al., 2002; Shapira et al., 2003; Dresbach et al., 2006; Cai et al., 2007; Maas et al., 2012). The α1 and Ca_v_ß subunits of the Ca_v_2.2 channel were biochemically suggested to reside on these compartments, but confirmation of their presence through microscopy was inaccessible due to technical limitations (Shapira et al., 2003). The other major transport organelle that has been identified is the Synaptic Vesicle Precursor (SVP), which carries SV markers including Synaptotagmin, Synaptophysin, and Rab3, and has not been shown to contain VGCCs (Okada et al., 1995; Maeder et al., 2014; Guedes-Dias et al., 2019; De Pace et al., 2020). SVP trafficking is primarily Kinesin-3 mediated (Okada et al., 1995). In addition to PTVs and SVPs, other transport organelles also likely exist. For example, mobile Neurexin puncta in axons do not colocalize with Bassoon but partially colocalize with other AZ proteins including CASK, RIM, and Mint, as well as the Ca_v_2.2 channel, suggesting the identity of a third AZ transport packet that has so far been minimally studied (Fairless et al., 2008).

One hypothesis for the bulk transport of AZ proteins (on PTVs) and SV proteins (on SVPs) is that this packaging allows robust and efficient maturation of new AZs into functional release sites. It has even been suggested that AZ and SV transport packets may be coordinated and comprehensive enough to be considered “pre-fabricated synapses” (Ahmari et al., 2000; Shapira et al., 2003; Tao-Cheng, 2007; Bury and Sabo, 2011; Vukoja et al., 2018). Indeed, comparisons of Piccolo and Bassoon abundance at synaptic and non-synaptic puncta suggest that only several PTVs are required to populate an AZ with its full complement of these proteins (Shapira et al., 2003; Tao-Cheng, 2007). Additionally, live imaging of movement and pausing suggests that although PTVs and SVPs represent separate organelles, they partially co-transport and share defined pause sites along axons (Bury and Sabo, 2011). Evidence from EM also points to bundled transport organelles, as Bassoon/Piccolo-positive aggregates of proteins, dense core vesicles, and smaller clear vesicles carrying SV markers can be seen in axons (Ahmari et al., 2000; Tao-Cheng, 2007). Light microscopy suggests VGCC subunits may colocalize with these organelle aggregates, but the lack of spatial resolution precludes determination of whether channels are present on PTVs, SVPs or a separate (and perhaps co-bundled) compartment (Ahmari et al., 2000).

Invertebrates were long thought to lack Piccolo and Bassoon (although *Drosophila* and *C. elegans* encode Piccolo-RIM homologs called Fife and Clarinet, respectively), calling into question whether they use PTV-like organelles to transport AZ proteins (Bruckner et al., 2017; Xuan et al., 2017). Like mammalian neurons, imaging in *Drosophila* axons revealed coordinated transport of some presynaptic proteins. The core AZ scaffold BRP partially co-transports with the SV protein Syt1. Interestingly, these transport packets colocalize with markers of non-degradative lysosomes and have thus been termed presynaptic lysosome-related vesicles (PLVs; Vukoja et al., 2018). Consistent with the role of Arl-8 in mediating synaptic protein and lysosome transport, *arl-8* mutations block trafficking of these PLV packets, resulting in their buildup in the cell soma. EM visualization of these stalled packets indicate they are ~60–70 nm in diameter and have variable electron densities resembling a mix of dense-core and clear transport vesicles as seen in mammals (Khatter et al., 2015; Vukoja et al., 2018). In mammalian neurons, the SV marker VGlut1 and the AZ marker Bassoon also co-transport with the lysosome marker Lamp1, and reductions in Arl-8 caused a buildup of Bassoon in the soma, suggesting this lysosomal transport mechanism also occurs in mammals (Vukoja et al., 2018). In contrast to the “pre-fabricated synapse” hypothesis, sequential steps of AZ assembly are clearly temporally and genetically separable in *Drosophila*, as some proteins populate AZs ahead of others and *rab3* mutations produce a sub-population of AZs that have only early AZ scaffolds but not late scaffolds or VGCCs (Fouquet et al., 2009; Graf et al., 2009; Owald et al., 2010; Böhme et al., 2016; Ghelani and Sigrist, 2018). Though a picture is emerging for the trafficking of core AZ scaffolds and SV components, how VGCCs traffic to synapses is still a major unknown. Determining if VGCCs travel in association with PTV/SVP aggregates or other post-Golgi vesicles, and which motor proteins mediate their transport, will require developing new tools to visualize VGCC trafficking in live neurons.

## The AZ clusters VGCCs

Once VGCCs arrive at the synaptic terminal, they are incorporated into an AZ (Figure 4 step 4). The AZ is a defined region of presynaptic membrane featuring a dense protein matrix that functions as a scaffold to cluster SVs near VGCCs for efficient Ca^2+^-mediated fusion (Südhof, 2012). The structure of the AZ scaffold differs between species and neuron types, but it is comprised of several conserved proteins (including RIM, RIM-binding protein, and ELKS/CAST) that help cluster VGCCs (Table 1; Figure 1; Zhai and Bellen, 2004). The interactome of Ca_v_2 channels has been analyzed in rodent brains using multi-epitope affinity purification and mass spectrometry, revealing a large cohort of ~200 proteins that interact with the channel (Müller et al., 2010). Multiple protein classes were identified, including known AZ scaffolds (CASK, RIM, RBP, Piccolo, etc) and other proteins that may regulate or function downstream of the channel. Given the multitude of proteins that bind VGCCs and contribute to their abundance and localization, a key question arises: which of these interactions is rate-limiting for VGCC accumulation? Identifying proteins that regulate VGCC abundance in a dosage-sensitive manner is critical, as these rate-limiting interactions may represent candidates for modulation during synaptic plasticity. The molecular constituents of the AZ and their roles in promoting VGCC clustering have been reviewed in depth (Dolphin and Lee, 2020; Gandini and Zamponi, 2022). Here we review the interactions of AZ proteins with VGCCs with a focus on distinguishing requirement versus rate-limiting roles.

### RIM and RBP

RIM-interacting molecule and RBP are central scaffolds that semi-redundantly regulate VGCC abundance at AZs (Han et al., 2011; Kaeser et al., 2011; Liu et al., 2011; Graf et al., 2012; Jung et al., 2015; Oh et al., 2021). RIM was identified through its interaction with the SV protein Rab3 (Wang et al., 1997), but it also interacts with multiple AZ-resident proteins including Ca_v_2 family VGCCs (*via* RIM’s PDZ domain), ELKS/CAST, RBP, Munc-13, and Liprin-α (Wang et al., 2000; Betz et al., 2001; Coppola et al., 2001; Ohtsuka et al., 2002; Schoch et al., 2002; Kiyonaka et al., 2007; Müller et al., 2010; Kaeser et al., 2011). Conditional knock-out of all PDZ-domain-containing *rim* genes from mammalian neurons results in ultrastructurally normal AZs with ~40% reduced Ca_v_2.1 channel abundance, similar to the partial loss of Ca_v_2 channels in *Drosophila* and *C. elegans rim* mutants (Koushika et al., 2001; Kaeser et al., 2011; Graf et al., 2012; Oh et al., 2021). Mouse *rim* mutants show a dramatic reduction in evoked release that is secondary to a decrease in presynaptic Ca^2+^ influx and fewer docked SVs (Han et al., 2011; Kaeser et al., 2011). RIM’s PDZ domain is required to rescue Ca_v_2.1 AZ abundance, while its RBP-binding sequences are required for normal [Ca^2+^]-dependence of release, indicating that both RIM’s direct interaction with Ca_v_2.1 and indirect interactions through RBP contribute to Ca_v_2.1 channel localization (Hibino et al., 2002; Kaeser et al., 2011).

RBPs-interacting molecule-binding proteins were identified through their binding interaction with RIMs, but they also directly bind VGCCs and Liprin-α (Wang et al., 2000; Hibino et al., 2002; Müller et al., 2010; Liu et al., 2011). RBPs role in setting VGCC abundance varies between neuron types and is semi-redundant with RIM. In *C. elegans*, *rbp* deletion alone did not change VGCC abundance at AZs, but *rim/rbp* double nulls had more severe VGCC depletion than either individual mutation (Kushibiki et al., 2019). Similarly, conditional deletion of both neuronally-expressed *rbp* genes in mammalian neurons did not alter VGCC abundance, but removing both RIM and RBP families at the calyx of Held resulted in a more severe (~75%) disruption in presynaptic Ca^2+^ influx (and in AZ ultrastructure) than deletions of RIMs alone, suggesting partially redundant roles for RIM and RBP in localizing VGCCs (Acuna et al., 2015, 2016). Though *Drosophila rpb* mutations do independently disrupt VGCC clustering (possibly downstream of a disorganized BRP scaffold), *rim/rbp* double heterozygotes have severely reduced release despite normal function in each individual heterozygote, further suggesting functional redundancy (Kittel et al., 2006; Fouquet et al., 2009; Liu et al., 2011; Müller et al., 2015; Bruckner et al., 2017; Ortega et al., 2018). Though RBP plays a secondary role to RIM in many systems, RBP does independently regulate VGCCs in hair cells, where *rbp* mutants display ~40% reduction in presynaptic Ca^2+^ influx and a similarly reduced level of synaptic Ca_v_1.3 immunofluorescence (Krinner et al., 2017).

These studies suggest that while RIM and RBP both bind to VGCCs, RIM is the dominant regulator of VGCC abundance at many synapses with partially redundant functions to RBP. This picture is complicated by *in vivo* structure–function studies at the calyx of Held, where Ca_v_2.1 C-terminal truncation constructs lacking the known RIM and RBP binding domains rescue presynaptic currents in *ca_v_2.1* conditional knockouts, suggesting these binding domains are dispensable for Ca_v_2.1 localization (Lübbert et al., 2017). This finding likely reflects redundancy in binding interactions that localize the channel to presynaptic membranes and may indicate that the known binding interactions of RIM to Ca_v_ß, or perhaps direct or indirect binding to another unknown site on the α1 subunit, provides an additional mechanism of interaction (Kiyonaka et al., 2007). Functional redundancy in localizing VGCCs presents a challenge in deciphering whether RIMs or RBPs play a dosage-sensitive role in fine-tuning VGCC AZ abundance. Future experiments testing whether RIM or RBP levels can be bidirectionally modulated to fine-tune VGCC abundance at AZs would provide insights into whether the abundance of these components rate-limits VGCC clustering.

### CAST/ELKS

In addition to RIM and RBP, the two semi-redundant CAST/ELKS family proteins (CAST and ELKS) are conserved core AZ scaffolds, initially discovered through biochemical analysis (Ohtsuka et al., 2002; Wang et al., 2002). In mammals, CAST/ELKS interacts directly with VGCCs, and other core AZ proteins including RIM, Munc13, and Liprin-α (Ohtsuka et al., 2002; Ko et al., 2003; Wang et al., 2009; Chen et al., 2011; Billings et al., 2012; Kiyonaka et al., 2012). Similarly, the *Drosophila* CAST/ELKS homolog BRP diverges from mammalian ELKS in its C-terminal domain but interacts with presynaptic Ca_v_2 channels through its ELKS-homologous N-terminal domain (Wagh et al., 2006; Fouquet et al., 2009). Despite their presence at synapses and direct interactions with VGCCs, the role of CAST/ELKS proteins in regulating VGCC abundance varies across systems. In mouse hippocampal synapses, conditional knockout of both *elks* genes after synapse formation resulted in a 30% decrease in presynaptic Ca^2+^ influx without any change in presynaptic VGCC abundance or synaptic ultrastructure (Liu et al., 2014). However, this manipulation was made after Ca_v_2 channels had already populated synapses, so whether this timeframe is long enough to see an ELKS-dependent effect on Ca_v_2 levels depends both on the AZ half-life of Ca_v_2 channels and the role of the ELKS-VGCC binding interactions. At mature (Ca_v_2.1-exclusive) and immature (Ca_v_2-mixed) mouse calyx of Held synapses, conditional knockout of *elks* in the *cast* null line caused mildly decreased Ca_v_1.3 abundance (Dong et al., 2018; Radulovic et al., 2020). In *C. elegans*, ELKS does not play a major role in clustering VGCCs at AZs (Deken et al., 2005; Oh et al., 2021). In contrast, the *Drosophila* CASK/ELKS homolog BRP plays a central role in forming the core of the AZ “T-bar” scaffold, promoting VGCC clustering and recruiting SVs to AZs (Kittel et al., 2006; Wagh et al., 2006; Fouquet et al., 2009). *Brp* mutants lack consolidated Ca_v_2 clusters and have a large decrease in evoked synaptic transmission (Kittel et al., 2006; Fouquet et al., 2009). Unlike mammalian CAST/ELKS which is not always required for AZ morphology and structure (Dong et al., 2018), *brp* null mutants lack the AZ “T-bar” (Kittel et al., 2006; Fouquet et al., 2009). Despite this requirement for BRP in clustering VGCCs at the AZ, BRP is not a rate-limiting regulator of VGCC abundance, as ~35% reductions in AZ BRP have no impact on VGCC abundance and do not change single AP-evoked SV release (Müller et al., 2015; Cunningham et al., 2022).

### Bassoon

The remaining core AZ proteins that are well characterized are Piccolo and Bassoon, Liprin-α, Syd-1, and Munc-13 (Cases-Langhoff et al., 1996; Tom Dieck et al., 1998; Fenster et al., 2000; Ackermann et al., 2015; Gundelfinger et al., 2015). Of these, Bassoon plays the most prominent role in VGCC localization, although Liprin-α is required for channel localization in *C. elegans* (Oh et al., 2021). Bassoon is a large multi-domain scaffolding protein that co-immunoprecipitates with Ca_v_ß and promotes VGCC clustering in some systems (Frank et al., 2010; Chen et al., 2011; Davydova et al., 2014). Binding between Bassoon and the VGCC-interaction partner RBP is important for recruiting Ca_v_2.1 (but not Ca_v_2.2) channels to hippocampal synapses (Davydova et al., 2014). At ribbon synapses in mammalian sensory neurons, the major AZ phenotype in *bassoon* mutants is loss of the ribbons (Dick et al., 2003; Khimich et al., 2005;Frank et al., 2010; Jing et al., 2013). At inner hair cell synapses, Ca_v_1.3 abundance is reduced even though some AZs have intact ribbons, indicating the *bassoon* VGCC-reduction phenotype is not completely downstream of ribbon loss (Frank et al., 2010; Jing et al., 2013). The Bassoon homolog Piccolo does not have an established role in promoting VGCC abundance, although it has been suggested to bind to VGCCs (Müller et al., 2010) and interacts with L-type VGCCs in pancreatic cells (Shibasaki et al., 2004). In contrast to the requirement of Bassoon for proper VGCC abundance and ribbon attachment at mammalian sensory synapses, Bassoon plays more minor roles in synaptic ultrastructure at mammalian central synapses (Altrock et al., 2003; Mukherjee et al., 2010). Although invertebrates were thought to lack Piccolo/Bassoon homologs, the Piccolo/Rim related proteins (Fife and Clarinet) were recently identified in *Drosophila* and *C. elegans*, respectively (Bruckner et al., 2017; Xuan et al., 2017). *Fife* mutants display a modest reduction in VGCC abundance at AZs (Bruckner et al., 2017). In summary, redundant interactions between the core AZ scaffolds (RIMs, RBPs, ELKS/CAST, and Bassoon) with each other and VGCCs provide a robust mechanism to ensure AZs are populated with VGCCs required to support synaptic transmission.

## The slot model for VGCC accumulation at AZs

A popular slot model of VGCC AZ abundance was originally proposed to explain several observations of competition among VGCCs for AZ localization in cultured hippocampal neurons with mixed Ca_v_2.1 and Ca_v_2.2 synapses (Cao et al., 2004). Overexpressing Ca_v_2.1 did not increase Ca_v_2.1-mediated release at synaptic terminals, suggesting the number of Ca_v_2.1 channels that localize to AZs is limited downstream of Ca_v_2.1 biosynthesis. Overexpression of mutant Ca^2+^-impermeant Ca_v_2.1 channels reduced the contribution of Ca_v_2.1 to total release, further indicating that mutant Ca_v_2.1 channels compete with their wildtype counterparts for AZ localization “slots” (Cao et al., 2004). Because whole cell (somatodendritic) Ca_v_2.1 currents were normal despite mutant channel overexpression, and were increased 5-fold by WT Ca_v_2.1 overexpression, the rate-limiting factor in AZ localization is proposed to be downstream of channel biosynthesis and surface expression (Cao et al., 2004). Additionally, Ca_v_2.2 influx was unaltered by Ca_v_2.1 overexpression, suggesting the existence of “Ca_v_2.2 specific slots” that cannot be occupied by Ca_v_2.1 (Cao et al., 2004). In a similar series of experiments, overexpressing Ca^2+^-impermeant Ca_v_2.2 reduced synaptic release, further indicating competition for saturated VGCC “slots” (Cao and Tsien, 2010). While Ca_v_2.2 overexpression failed to increase total presynaptic release, Ca_v_2.2 channels could displace Ca_v_2.1 channels, suggesting “Ca_v_2.1-preferring slots” can accommodate Ca_v_2.2 under conditions of Ca_v_2.2 excess (Cao and Tsien, 2010).

Three key elements define the slot model for limiting VGCC accumulation at AZs. First, Ca_v_2.1/Ca_v_2.2 mixed synapses are proposed to have “Ca_v_2.1-preferring slots” and “Ca_v_2.2 specific slots” that limit the number of VGCCs at the AZ. Second, “slots” are typically saturated, supported by the observation that channel overexpression does not increase AZ channel levels (Cao et al., 2004). Third, “slots” may not represent a limited number of rigid locations at the AZ where VGCCs are physically tethered, but may instead include competition for binding partners at any stage of VGCC localization all the way from axonal trafficking to channel incorporation or stabilization at AZs. Since Ca_v_2.1 and Ca_v_2.2 overexpression increased cell body Ca^2+^ currents, the competition for rate-limiting binding partners (“slots”) are proposed to be downstream of ER exit and cell surface expression (Cao and Tsien, 2010). The slot model predicts Ca_v_2.2 channels should compensate for loss of Ca_v_2.1 expression, whereas Ca_v_2.1 channels should be unable to occupy Ca_v_2.2-specific slots in Ca_v_2.2 mutants. Indeed, Ca_v_2.2 channels partially compensate in *ca_v_2.1* knockout mice at the mature calyx of Held, but Ca_v_2.1 does not increase in *ca_v_2.2* mutant hippocampal neurons (Kim et al., 2001; Inchauspe et al., 2004; Ishikawa et al., 2005; Jeon et al., 2007). Additionally, Ca_v_2 α1 subunit overexpression in dissociated rat neurons and *Drosophila* NMJs does not increase Ca_v_2 levels at synapses, providing further support for a competition model (Hoppa et al., 2012; Cunningham et al., 2022).

Despite evidence supporting the slot model, its predictions partially fail at the calyx of Held. At immature (Ca_v_2.1/Ca_v_2.2 mixed) and mature (Ca_v_2.1 exclusive) calyx neurons, Ca_v_2.1 overexpression increased Ca_v_2.1 number at AZs, indicating that if Ca_v_2.1 slots exist, they are not saturated at this synapse (Lübbert et al., 2019). However, some evidence of competition was still observed, as Ca_v_2.1 overexpression outcompeted Ca_v_2.2 channels in the immature calyx. These data support an alternative model where Ca_v_2.1 channels are not saturated at AZs, and Ca_v_2.2 slots are Ca_v_2.2-preferred rather than Ca_v_2.2-specific (Lübbert et al., 2019). Contrasting findings in hippocampal vs. calyx of Held neurons could be due to several factors. VGCC regulation could differ between cultured neurons vs. *in vivo* neurons embedded in native circuitry. In addition, rules for mixed synapses may differ and change during development. Finally, previous studies used human Ca_v_2.1 and Ca_v_2.2 overexpression in mouse neurons. Even though these constructs rescued their respective knockouts, human and mouse VGCCs could differ in their regulation (Cao et al., 2004; Cao and Tsien, 2010). Further experiments are needed to define which aspects of the slot model represent general principles versus synapse-specific regulation that reflect neuronal diversity.

Several important questions still need to be addressed in the classical slot model for AZ VGCC abundance. If slots exist, what do they physically represent? Is the slot mechanism implemented locally at AZs (by limiting incorporation or retention of channels) or upstream of AZ localization, perhaps through limited binding to axonal trafficking partners? The key criterion for identifying a protein that regulates competition is that the level of that protein should affect VGCC AZ abundance in a dosage sensitive manner. The conserved AZ scaffold proteins are attractive candidates for locally mediating slots at the AZ. *Drosophila* BRP is well situated to be a slot protein, as it binds directly to the Ca_v_2 channel and is required for channel accumulation and stabilization at AZs (Fouquet et al., 2009; Ghelani et al., 2022). However, reductions in AZ BRP abundance have no effect on AZ VGCC levels, indicating this protein is likely not a rate-limiting regulator of VGCCs at AZs (Cunningham et al., 2022). RIMs and RBPs initially were compelling candidates for a slot protein because they both bind to VGCCs and are required (albeit redundantly at different synapses) for VGCC localization (Han et al., 2011; Kaeser et al., 2011; Liu et al., 2011; Graf et al., 2012; Jung et al., 2015; Oh et al., 2021). Additionally, RIM interacts stoichiometrically with Ca_v_2 (Kaeser et al., 2011; Oh et al., 2021). However, in mammalian central synapses and *Drosophila* NMJs, loss of RIM and RBP binding to the C-terminal of VGCCs did not reduce channel AZ localization (Schneider et al., 2015; Lübbert et al., 2017; Ghelani et al., 2022). Indeed, several studies reported the lack of RIM and RBP interactions actually promotes channel stability at AZs, opposite to what would be expected for a protein functioning as a VGCC slot interactor (Schneider et al., 2015; Ghelani et al., 2022). Bassoon has also been proposed to contribute to defining Ca_v_2.1 slots, as the Bassoon-RBP interaction recruits Ca_v_2.1 (but not Ca_v_2.2) channels to hippocampal synapses (Davydova et al., 2014). However, Bassoon does not appear to regulate VGCC abundance in all neurons (Altrock et al., 2003; Mukherjee et al., 2010). The α2δ subunit is another possible “slot” protein, as it plays a dosage-sensitive role in promoting AZ VGCC abundance. Overexpression of α2δ leads to increased VGCC levels at synapses, while heterozygous mutations in this subunit moderately decrease Ca_v_2 levels at AZs (Hoppa et al., 2012; Cunningham et al., 2022). A role for α2δ as the slot factor would likely be in its capacity as a VGCC trafficking regulator, as *α2δ* mutants actually show increased Ca_v_2 retention at synapses, arguing against AZ-localized α2δ as the regulator of slot number (Cunningham et al., 2022). Another possibility is the slot interaction is lipid-mediated, as cholesterol has been shown to restrict VGCC domain size at AZs in photoreceptors (Mercer et al., 2011). Deciphering which molecules and binding interactions are rate limiting for channel localization to AZs is an important goal but is complicated by functional redundancy and other potential compensatory mechanisms.

## VGCCs are mobile within the AZ

Characterizing mobility of VGCCs within the AZ is a topic of interest, given positional coupling of VGCCs and docked SVs is a major determinant of *P_r_* (Bucurenciu et al., 2008; Eggermann et al., 2011; Chen et al., 2015). VGCC mobility within the AZ could represent a fast method of *P_r_* regulation by altering the channel’s coupling distance to docked SVs. The idea that VGCCs occupy defined spots at the AZ arose from studies at the frog NMJ, where freeze fracture EM showed an array-like organization of particles, generating questions of whether these particles represent statically arranged VGCCs (Heuser et al., 1974; Pumplin et al., 1981; Cohen et al., 1991; Harlow et al., 2001). However modeling and experimental estimation of VGCC number at the frog NMJ suggests not all of these intramembrane particles can be channels, and Ca_v_-immunogold EM reveal a less orderly, but non-randomly clustered, array of VGCCs (Luo et al., 2011; Holderith et al., 2012; Althof et al., 2015; Miki et al., 2017; Lübbert et al., 2019; Eguchi et al., 2022). The model of an orderly array of VGCCs is also at odds with more recent evidence from *in vivo* tracking of single VGCCs at synapses, showing that a fraction of AZ-resident VGCCs are mobile within a defined region of membrane, with low exit of channels from the AZ area (Mercer et al., 2011, 2012; Thoreson et al., 2013; Schneider et al., 2015; Figure 4; step 5).

At photoreceptor ribbon synapses of the salamander retina (populated with L-type VGCCs), quantum dots tagged to the extracellular α2δ-4 subunit of the channel revealed mobility within a defined ~0.18 μm^2^ region of presynaptic membrane under the ribbon (Mercer et al., 2011, 2012). In addition to baseline VGCC movements, SV fusion briefly displaced VGCCs toward the outer rim of the membrane region (Mercer et al., 2011). In both photoreceptors and bipolar cells, actin restricts the size of the VGCC-mobile area, consistent with studies showing actin disruption promotes VGCC internalization (Cristofanilli et al., 2007; Mercer et al., 2011; Thoreson et al., 2013). Cholesterol levels also regulate VGCC mobility within photoreceptor synapses, as cholesterol depletion widened the VGCC-mobile area and reduced *P_r_* without altering Ca^2+^ influx, suggesting mobility may be regulated to tune VGCC-SV coupling distances (Mercer et al., 2011, 2012). Additionally, movement of an open VGCC could spread Ca^2+^ over a larger area, reducing the effective peak Ca^2+^ concentration compared to influx from stabilized VGCCs. This “smearing” factor may be especially relevant at highly sensitive synapses in photoreceptors or bipolar cells where the opening of only one or a few VGCCs is sufficient to trigger SV fusion (Jarsky et al., 2010; Bartoletti et al., 2011; Kim et al., 2013). Though modeling suggests the expanded VGCC-domain size is sufficient to account for decreased release, the effect of cholesterol depletion on other proteins involved in SV fusion cannot be ruled out (Mercer et al., 2012). These tracking experiments provide insights into the mobility of VGCCs, but it is unclear if α2δ-4-QDot tagging is a robust proxy for VGCC α1 subunit localization and mobility, as α2δ subunits regulate synapse development independent of their canonical position as VGCC subunits and can localize to synaptic terminals independent of the VGCC (Kurshan et al., 2009; Dolphin, 2018; Held et al., 2020). Furthermore, studies in hippocampal cultured neurons suggest association of VGCC α1 and α2δ is dynamic, with α2δ showing more mobility than the VGCC α1 subunit (Voigt et al., 2016).

Direct single-particle tracking of VGCC α1 subunits in mammalian cultured neurons have circumvented this caveat. SptPALM imaging of cytoplasmic mEOS2-tagged Ca_v_2.1 and Ca_v_2.2 channels in hippocampal neurons revealed the population of VGCCs within clusters is comprised of a mobile fraction (~60% of channels) and a smaller immobile fraction (Schneider et al., 2015). Channel mobility was largely confined within individual synapses, exhibited transient (~80 ms) confinement within synaptic nanodomains, and was similar for both Ca_v_2.1 and Ca_v_2.2, in contrast to mEOS2-tagged Syntaxin-1A (Schneider et al., 2015; Heck et al., 2019). Interestingly, reducing intracellular Ca^2+^ using BAPTA increased the fraction of immobile VGCCs, hinting at a possible mechanism to modulate VGCC mobility during plasticity (Schneider et al., 2015). In addition to regulation by Ca^2+^ buffering, VGCC mobility is activity-dependent, as blocking action potentials or postsynaptic glutamate receptors results in channel stabilization (Heck et al., 2019). Scaffold-channel interactions also regulate mobility; though surprisingly, the Ca_v_2.1 splice variant lacking a C-terminal exon that encodes both RIM and RBP binding domains displays decreased mobility and supports more efficient SV release (Heck et al., 2019). These α1 tracking experiments in cell culture represent exciting steps forward in understanding channel mobility, as they reveal direct localization of the channel without relying on α2δ as a localization proxy. However, whether the lack of *in vivo* connections and a native synaptic environment abnormally influences channel mobility is currently unclear. Similar single-VGCC tracking experiments are currently being performed *in vivo* at *Drosophila* NMJs, where VGCCs appear to undergo high intra-AZ mobility as well (Ghelani et al., 2022). In addition, insights into longer-term VGCC mobility at *Drosophila* NMJs using photoconvertible Cac channels reveal they do not appear to laterally diffuse between neighboring AZs over multiple days, suggesting low inter-AZ movement despite high intra-AZ mobility (Cunningham et al., 2022).

## VGCC internalization from AZs

The lifetime of surface expression for transmembrane proteins varies widely and is regulated in part by re-internalization through endocytosis (Figure 4; step 6). Most endocytosis is through a relatively slow Clathrin-mediated process, with adaptor proteins concentrating cargo and recruiting Clathrin, which assembles to deform the membrane into a pit. Subsequently, a burst of Actin to budding endocytic vesicles and membrane pinching by the GTPase Dynamin completes the endocytic process (Kaksonen and Roux, 2018). Faster Clathrin-independent modes of endocytosis have also been described at synapses. Bulk and ultrafast endocytosis are thought to quickly retrieve synaptic membrane after SV fusion (Watanabe and Boucrot, 2017). In addition, fast Endophilin mediated endocytosis (FEME) can be initiated to internalize specific membrane proteins, including some G Protein-Coupled Receptors (GPCRs; Moo et al., 2021). GPCRs are inhibited by their own agonist-stimulated endocytosis, a process which is canonically initiated by the binding of endocytic adaptor proteins of the Arrestin family, but that can also proceed through non-canonical pathways (Moo et al., 2021; von Zastrow and Sorkin, 2021). Receptor Tyrosine Kinases (RTKs) are also endocytosed after ligand binding, with internalization initiated either though RTK ubiquitination or binding to Clathrin-adapter proteins (von Zastrow and Sorkin, 2021). AMPA Receptors are glutamate-gated cation channels that mediate most excitatory neurotransmission in the mammalian CNS and their internalization regulates synaptic strength during several forms of synaptic plasticity (Citri and Malenka, 2008; Hastings and Man, 2018). AMPA Receptors can dissociate from scaffolds within the postsynaptic density and move into endocytic zones where they associate with Clathrin adaptor proteins and become internalized. In contrast to GPCRs, RTKs, and AMPA receptors, little is known about the role of internalization in VGCC regulation at AZs. How big of a role does VGCC internalization play in regulating synaptic strength? What regulates VGCC internalization and what molecular pathways facilitate this process? Does channel endocytosis occur within AZs or elsewhere on the presynaptic membrane?

Some evidence for GPCR-regulated VGCC internalization has come from studies of Ca_v_2.2 channels in cultured neurons and DRG neurons involved in pain signaling (Bourinet et al., 2014). In this circuit, the GPCR opioid receptor (ORL1) forms a complex with Ca_v_2.2 channels and its activation *via* the agonist nociceptin results in ORL1/Ca_v_2.2 complex internalization (Beedle et al., 2004; Altier et al., 2006). This internalization can be directly visualized using GFP-tagged Ca_v_2.2 α1 subunits and red-tagged ORL receptors. Upon ORL activation, Ca_v_2.2 and ORL exclusively colocalize in intracellular puncta that label with a lysosomal marker (Altier et al., 2006). In acutely dissociated DRG neurons, Ca_v_2.2 channels are internalized following nociceptin exposure, leading to a decrease in Ca_v_2.2-mediated Ca^2+^ influx (Altier et al., 2006). Though lysosome marker colocalization suggests internalized channels may be degraded, the fate of these channels is unknown. Agonist washout results in loss of intracellular VGCCs after several hours, but whether channels were returned to the surface or targeted for degradation is unclear (Altier et al., 2006). Dopamine (DA) receptors are another family of GPCRs that regulate VGCCs in the mammalian CNS, and DA receptors can promote internalization of Ca_v_2.2 through direct protein–protein interactions (Kisilevsky et al., 2008; Kisilevsky and Zamponi, 2008). Along with GPCR regulation, the Actin cytoskeleton plays a role in regulating presynaptic VGCC internalization in some systems (Furukawa et al., 1997; Cristofanilli et al., 2007; Mizuno et al., 2010; Tseng et al., 2017).

Studies of molecular mechanisms of VGCC internalization in cell culture is facilitated by molecular and imaging access, but *in vivo* experiments are required to understand the timescales and patterns of VGCC internalization at native synapses. Do AZ-localized VGCCs become internalized through similar pathways? How long do VGCCs remain at the presynaptic membrane and how is their internalization regulated? Animal-wide isotopic labeling has been employed as a high-throughput strategy for measuring protein half-lives *in vivo* (Price et al., 2010; Fornasiero et al., 2018; Heo et al., 2018). This approach can measure degradation rates of newly synthesized proteins across the entire proteome, but has limited spatial resolution to measure turnover in specific compartments or individual neuronal populations. Given degradation of VGCCs can occur in the biosynthetic pathway before channels reach the synapse, whole-brain turnover measurements may not accurately reflect rates of AZ-localized VGCCs dynamics. Despite these drawbacks, it is worth noting that VGCCs display a half-life of around 8 days when assayed by isotopic labeling (Fornasiero et al., 2018).

Studies of AZ-resident VGCC half-life and turnover have also been performed at the *Drosophila* NMJ, a synapse with hundreds of AZs that are individually resolvable by conventional light microscopy in intact animals, allowing multi-day experiments using intravital imaging (Figures 2B,C). Red-to-green photoconversion of endogenously Maple-tagged Cac (the sole VGCC mediating synaptic transmission in flies) allowed measurements of Cac removal from AZs over a multi-day period. On average, 30% of photoconverted Cac signal intensity was lost from AZs over 4 days, indicating turnover plays an important role in regulating the abundance of the channel at AZs (Figures 2D–G; Cunningham et al., 2022). This 30% loss over 4 days predicts a half-life of about 8 days, consistent with isotopic labeling measurements of VGCC stability (Fornasiero et al., 2018; Cunningham et al., 2022). Measurements of new Cac delivery at individual AZs indicates turnover contributes to a leveling-off of Cac abundance at mature AZs. Furthermore, Cac loss from AZs is predicted to occur primarily through re-internalization of the channel, as lateral transfer of Cac channels was not observed (Cunningham et al., 2022). In mutants with either reduced levels of α2δ or reduced levels of the α1 subunit, turnover was reduced, indicating new channel delivery regulates channel turnover at this synapse rather than a fixed VGCC lifespan (Cunningham et al., 2022).

## Presynaptic VGCCs: Beyond evoked neurotransmission

Aside from the canonical role of presynaptic VGCCs as mediators of evoked neurotransmission, non-AZ resident VGCCs can regulate other Ca^2+^-dependent aspects of presynaptic function, including SV endocytosis and presynaptic plasticity. At presynaptic terminals, Ca^2+^-dependent endocytosis immediately follows SV fusion (Hosoi et al., 2009; Wu et al., 2009, 2014). Temporal coordination between exo- and endocytosis ensures prompt recycling of SVs after fusion, and maintains presynaptic membrane homeostasis (for a detailed review of presynaptic exo-endocytic coupling, see Wu et al., 2014; Maritzen and Haucke, 2018). Ca^2+^ influx through VGCCs has been proposed to mediate this coupling (Wu et al., 2009, 2014; Xue et al., 2012; Krick et al., 2021). In addition to SV fusion and endocytosis, Ca^2+^ signaling through VGCCs can contribute to short term plasticity without altering baseline *P_r_*, reflecting functional separation between VGCC subtypes within the presynaptic membrane (Jensen and Mody, 2001; Dietrich et al., 2003; Krick et al., 2021).

Given multiple processes—including neurotransmission, endocytosis, and plasticity—are controlled by VGCC-dependent Ca^2+^ signaling within a relatively small area, how are these Ca^2+^ signals separated to avoid crosstalk? Precise positioning of different VGCC subtypes within subdomains of the presynaptic membrane is one mechanism by which synapses can reduce crosstalk. This spatial separation of distinct VGCC populations in the presynaptic terminal is illustrated at the *Drosophila* NMJ, where the sole Ca_v_2 channel (Cac) localizes to AZs and mediates neurotransmission, while the Ca_v_1 channel (Dmca1D) localizes to non-AZ domains within the presynaptic membrane and regulates Ca^2+^-dependent endocytosis and short-term plasticity (Krick et al., 2021). In addition to the distinct localizations of these channel types, cytosolic Ca^2+^ buffers and active extrusion of Ca^2+^ by the PMCA pump further reduces crosstalk between Ca_v_1 and Ca_v_2 signaling (Krick et al., 2021). Similar to AZ-resident VGCCs, the mechanisms that regulate the abundance and subcellular localization of other VGCC populations within the presynaptic membrane are unknown.

## Modulation of VGCC abundance during plasticity: Insights from *Drosophila*

Voltage-gated Ca^2+^ channels are key regulators of presynaptic *P_r_*, placing them in a prime position to be targeted by plasticity pathways that modulate synaptic strength (Augustine et al., 1985; Borst and Sakmann, 1996; Wang et al., 2008; Bartoletti et al., 2011; Ariel et al., 2012; Sheng et al., 2012; Newman et al., 2022). Indeed, acute modulation of VGCC activation, inactivation, and conductance have all been shown to contribute to various presynaptic plasticity pathways (Nanou and Catterall, 2018). More recently, studies at the *Drosophila* NMJ indicate plastic regulation of channel abundance and mobility at the presynaptic membrane can also occur. Due to robust genetic, imaging, and electrophysiological approaches that enable studies of individual AZs *in vivo*, this model has emerged as a key system for characterizing how presynaptic plasticity impinges on the abundance and mobility of AZ components. Indeed, changes in the abundance and organization of VGCCs and the AZ scaffold have been reported at the NMJ during expression of acute and chronic forms of plasticity.

When postsynaptic Glutamate Receptors are blocked acutely with a toxin or chronically *via* genetic mutations at *Drosophila* NMJs, the decrease in postsynaptic responsiveness to neurotransmitter (quantal size) triggers a compensatory upregulation of presynaptic *P_r_*. Increased SV fusion precisely offsets the reduction in quantal size, homeostatically preserving overall synaptic strength. This homeostatic synaptic potentiation (HSP) can happen strikingly fast, occurring on the scale of minutes after application of a Glutamate Receptor toxin (Frank et al., 2006, 2009; Müller and Davis, 2012; Frank, 2014). The Cac channel is mechanistically implicated in HSP plasticity. Ca^2+^ imaging directly demonstrates an increase in presynaptic Ca^2+^ influx during HSP, and hypomorphic mutations in *cac* block potentiation of Ca^2+^ influx and homeostatic potentiation (Frank et al., 2006; Müller and Davis, 2012). Additionally, the extracellular Cac subunit α2δ is required for HSP, independent of its effect on baseline Ca^2+^ influx (Wang et al., 2016). An opposing form of presynaptic homeostatic plasticity is homeostatic synaptic depression (HSD). When quantal size is chronically increased through overexpression of the SV glutamate transporter VGlut, HSD compensates by reducing presynaptic *P_r_*. Imaging of Ca^2+^ influx and AZ Cac-GFP abundance demonstrates this form of plasticity also targets the Cac channel by decreasing its abundance at AZs (Gaviño et al., 2015). Together these findings suggest Ca^2+^ influx through VGCCs is modulated bidirectionally to influence *P_r_* during multiple forms of presynaptic plasticity.

While initial evidence for Cac involvement in HSP did not resolve whether channel properties or abundance were altered to trigger increased Ca^2+^ influx, several studies suggest AZ Cac abundance may increase during this form of potentiation (Gratz et al., 2019; Ghelani et al., 2022). Similarly, BRP puncta observed in AZ rings increased in number during HSP (Hong et al., 2020). Although these studies suggest elevated levels of BRP and Cac, studies employing STORM imaging indicate the increased fluorescent intensity is secondary to compaction of AZ material that occurs during plasticity rather than increases in protein content across AZs (Mrestani et al., 2021). Work in the *Drosophila* CNS indicates Cac transcription may also be a target for certain forms of presynaptic potentiation. In the *Drosophila* CNS, Kenyon Cell neurons form boutons along compartmentalized regions of the mushroom body to drive associative learning. Monitoring of presynaptic Ca^2+^ during behavior reveals compartment-specific modulation of Ca^2+^ influx along Kenyon Cell axons during learning that is mediated by neuromodulatory neuron dopamine release and presumed GPCR-mediated silencing of VGCC function (Bilz et al., 2020). Although reducing VGCC biosynthesis by modest levels does not alter baseline transmission at these synapses, the same manipulation impairs presynaptic potentiation, indicating Cac biosynthesis becomes rate-limiting during certain forms of presynaptic plasticity (Stahl et al., 2022). Together, these studies suggest VGCC abundance, location, and mobility at AZs may represent important targets for fine-tuning of presynaptic output.

## Conclusion and future directions

Pathways regulating the surface abundance of presynaptic VGCCs, including progression through the biosynthetic pathway, transport to the synapse, stabilization and mobility at AZs, and turnover by endocytosis, have emerged as important mechanisms to set baseline synaptic strength and as potential targets to change output during plasticity. Despite the importance of VGCC dynamics and regulation, many questions remain unsolved. In particular, identifying which VGCC-regulatory components are rate-limiting in setting channel abundance at AZs will provide insights into the fine-tuning and regulation of channel surface expression. Additionally, the precise mechanisms and timescales of channel delivery and turnover at individual presynaptic AZs are still unclear, precluding a clear understanding of how delivery and recycling modulate synaptic development and presynaptic strength in growing circuits.

## Author contributions

All authors listed have made a substantial, direct, and intellectual contribution to the work and approved it for publication.

## Funding

The authors’ work has been funded by NIH grants NS40296, NS117588, and MH104536 and the JPB Foundation.

## Conflict of interest

The authors declare that the research was conducted in the absence of any commercial or financial relationships that could be construed as a potential conflict of interest.

## Publisher’s note

All claims expressed in this article are solely those of the authors and do not necessarily represent those of their affiliated organizations, or those of the publisher, the editors and the reviewers. Any product that may be evaluated in this article, or claim that may be made by its manufacturer, is not guaranteed or endorsed by the publisher.
